# Be careful with small supernumerary marker chromosomes!

**DOI:** 10.3389/fgene.2023.1269679

**Published:** 2023-10-10

**Authors:** Laura Rodríguez

**Affiliations:** Genetic Laboratory AbaCid, HM Hospitales, Hospital Universitario HM Sanchinarro, Madrid, Spain

**Keywords:** sSMC, centromere, misdivision, heterochromatin, euchromatin. genetic counseling

Small supernumerary marker chromosomes (sSMC) are defined as small structurally abnormal chromosomes whose origins cannot be characterized by conventional techniques but require molecular technology for their correct characterization. sSMCs are equal to or smaller in size than a chromosome 20 of the same metaphase spread. They are constituted by euchromatin (genetic material enriched with genes) and/or heterochromatin (gene-poor regions) material with a primary constriction (centromere). They can present with three possible shapes: inverted duplicated-shaped sSMC (“inv dup”), ring-shaped sSMC (“r”), or centric minute-shaped sSMC (“min”). According to the ChromosOmics-Database, the most frequently observed sSMC in terms of shape are “inv dup” sSMC (63%), followed by “min” sSMC (26%) and “r” sSMC (11%) (Liehr, 2023). Less is known about how sSMC are formed, as there are many questions about why, when, or how sSMC evolve during gametogenesis or embryogenesis. What seems clear is that a combination of one or more unusual events happening during gametogenesis or embryogenesis are related to these events, as well as the presence of a possible trisomic rescue.

sSMC have been described to derive from all human chromosomes, although 86% of the described sSMC are derived from acrocentric chromosomes, of which 50% derive from chromosome 15. The frequency of sSMC in the general population is estimated to be around 0.044%, although if we refer specifically to those prenatally diagnosed cases, their frequency can increase up to 0.075%. Likewise, sSMC originating “*de novo*” represent 70% of the total, compared with 30% which are inherited. These are often derived from maternal meiosis I/II errors, trisomic/monosomic rescue, or fertilization errors (Bartels et al., 2003; Hochstenbach et al., 2016). Around 70% of carriers of an sSMC are phenotypically normal individuals, while the remaining 30% will have some associated clinical repercussions. In fact, among sSMC diagnosed prenatally, the risk associated with an abnormal phenotype has been estimated to be around 13% (Liehr, 2023), although this risk has been redefined to 7% when derived from chromosomes 13, 14, 21, or 22, and 28% when derived from the rest of the autosomal chromosomes. sSMC have also been associated with fertility problems; in fact, in infertile couples, the frequency of sSMC carriers is around 0.125%, being 7.5 times higher prevalence of this in males than in females (Liehr, 2023). Furthermore, if we refer specifically to patients with some type of intellectual disability, the frequency of sSMC rises to 0.288%. It is also important to remember that these frequencies maintain no significant differences between the diverse ethnicities (Liehr, 2023).

Nonetheless, this great phenotypic variability associated with sSMC is nothing more than the result of the expression of the chromosome material contained in said sSMC, either heterochromatin (centromere and/or satellites) and/or euchromatin. Likewise, the phenotypic repercussion of each sSMC will depend on several aspects, such as: 1) sSMC can be derived from any chromosome and could be combined with other chromosomal changes; 2) the amount and function of genes contained in the sSMC (generally, the larger the sSMC, the larger amount of euchromatin and increased risk of phenotypic impact. In fact, two visibly identical sSMC originating from the same chromosome could differ in size or in content of euchromatic material and/or genes); 3) the presence in the same individual of different shapes of the same sSMC 4) several sSMC could be present in the same patient; 5) the degree of mosaicism of the sSMC (many sSMC are mitotically unstable so the number of cells or tissues bearing sSMC varies); and lastly 6) the increased risk of uniparental disomy (UPD) of the homologous chromosomes from which such sSMC is derived. In fact, several possible mechanisms related to the formation of UPD have been described associated with the presence of sSMC: 1) trisomic functional rescue (heterodisomy UPD); 2) postzygotic reduplication (isodisomy UPD); 3) post zygote formation error *1. Chromosome duplication or *2. Chromosome break (isodisomy UPD); and/or 4) complementation (heterodisomy UPD) (Kotzot, 2002; Liehr, 2010; Li et al., 2020; Liehr, 2023).

For all these reasons described, once an sSMC is found, usually by conventional cytogenetic banding analysis which is weak for identifying sSMC origins, the immediate procedure is to apply further analysis as FISH (Fluorescence *in situ* hybridization), Array-CGH (comparative genomic hybridization), and/or next-generation sequencing (NGS) for correct sSMC characterization. FISH is a directed technique (probe-depended), an approach that allows nucleic acid sequences to be analyzed in nuclei or in metaphases chromosomes. There are one color FISH probes from all chromosomes, as well as two-color, three-color, or multicolor FISH probe sets which are essential in routine cytogenetics analysis for sSMC characterization. Nowadays, array-CGH is widely used since it provides reliable information regarding copy number variations (CNVs), such as microdeletions and microduplications from all genomes, but it fails to detect balanced anomalies. Finally, NGS is a very novel technique of recent implementation with a much higher resolution capacity.

Once the sSMC is prenatally or postnatally found (usually by conventional cytogenetic), it is a priority to determine if it is “*de novo*” or inherited, and in all cases, it is essential to analyze other direct relatives to build a family tree of sSMC carriers, whether they do or do not present clinical manifestations. Simultaneously, the application of the above described techniques should be applied for sSMC characterization (morphology, content, and UPD). Thus, the combination of all this information will allow for specific searches of similar literary cases, which will guide the carrier prognosis (https://cs-tl.de/DB/CA/sSMC/0-Start.html, https://www.deciphergenomics.org/, https://www.ncbi.nlm.nih.gov/). This becomes much more complicated in a prenatal context, where it is also essential to carry out a high-resolution fetal ultrasound that allows for more accurate diagnosis of possible fetal anomalies for correct genetic counseling (Liehr, 2023).

**FIGURE 1:**
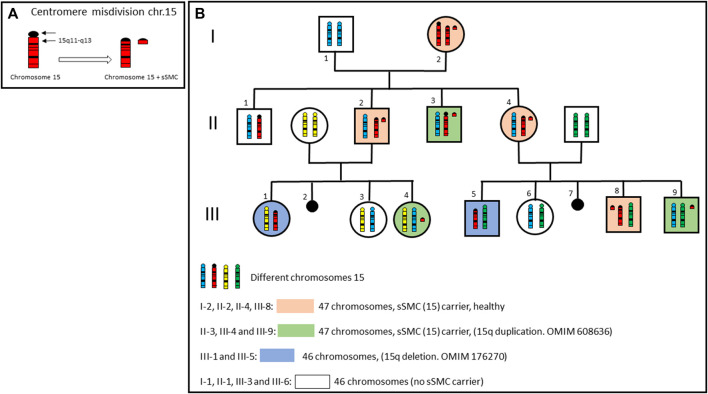
**(A)** Centromere misdivision of a chromosome 15. **(B)** Genealogy of a family with an sSMC derived from chromosome 15 produced by centromere misdivision. Only chromosomes 15 are showed in each member of the family. Individuals carrying sSMC, may or may not be chromosomally balanced, depending on which, they may or may not present clinical manifestations.

After everything described above, it seems evident that the finding of an sSMC carries an excess of chromosomal material, but it is important to note that on some occasions, it is the sSMC itself which confers balance to the carrier individual (Baldwin et al., 2008). This type of sSMC appears when an incorrect centromeric division (centromere misdivision, proposed by McClintock, B) occurs, resulting in a chromosome break centromere with either a short (p) or long (q) arm piece of the same chromosome involved (Figure 1B). These sSMCs are small fragments of a chromosome which carry a part of the euchromatin of said chromosome. Therefore, its presence is essential to achieve a balanced karyotype. It is crucial to consider this possibility, given that in the same family there may be carriers of sSMC (with 47 chromosomes) phenotypically normal and patients with an apparently normal karyotype (with 46 chromosomes) and partial deletion of the chromosome pericentromeric region involved in the misdivision. In Figures 1A, C, C genealogy of a family with an sSMC derived from chromosome 15 produced by centromere misdivision is shown. The individuals carrying the sSMC may or may not be chromosomally balanced, depending on which, they may or may not show clinical manifestations (OMIM 608636 and OMIM 176270).

In conclusion, it should be noted that the term “sSMC carrier” does not always mean excess of genetic material and that, therefore, we should all “be careful” when a sSMC is found, either prenatally or postnatally. Consequently, in order to offer a correct genetic counseling to the family, all family members should be tested for carriers of the correctly characterized sSMC.
